# Diet Quality and Nutritional Assessment of Menus Served in Spanish Defense Ministry Preschool Canteens

**DOI:** 10.3390/nu17040661

**Published:** 2025-02-12

**Authors:** Diego Lozano, María Abenoza, Susana Bayarri, Regina Lázaro

**Affiliations:** 1 Servicio de Seguridad Alimentaria y Salud Pública, Centro Militar de Veterinaria de la Defensa, C/Darío Gazapo 3, 28025 Madrid, Spain; dlozben@et.mde.es; 2 Instituto Agroalimentario de Aragón—IA2 (Veterinary Faculty, Universidad de Zaragoza—CITA), 50013 Zaragoza, Spain; mabenoza@unizar.es (M.A.); rlazaro@unizar.es (R.L.)

**Keywords:** preschool education centers, menu, nutritional assessment, diet quality, frequency, food groups, dietary recommendations

## Abstract

**Background/Objectives**: A healthy diet in early childhood has been shown to be one of the essential mainstays for the development of children. Few studies have been conducted on preschool canteens, despite the fact that they play a crucial role. Our study aimed to assess the dietary and nutritional quality of the menus served in Spanish Defense Ministry preschool education centers (1–3 years old) and to ascertain their compliance with dietary and nutritional recommendations. We also evaluated the influence of the canteen management model. **Methods**: Our cross-sectional observational study was carried out on the 448 menus offered in the 25 centers (operating under two management models: direct and contracted) of the Spanish Ministry of Defense during a school year all across the Spanish territory. **Results**: Under the contracted management model, the frequency of rice was low, as well as that of salads, eggs, fish, and fruit. The rotation within the fish group did not comply with recommendations, showing a shortage of oily fish. The menus’ energy values were correct, but the energy profile in the contracted management menus was higher than recommended in protein and fat while being lower in carbohydrates. The lipid profile was adequate. Values were high in cholesterol, simple sugars, fiber, vitamin K, selenium, potassium, and sodium; they were deficient in omega-3 fatty acids, vitamin D, iodine, and zinc. **Conclusions**: These results could help to establish special references or limits for the Spanish preschool population.

## 1. Introduction

The World Health Organization (WHO) considers childhood obesity one of the most serious public health problems of the 21st century. Approximately 60% of obese and overweight children tend to remain so in adulthood and are more likely to develop diseases such as diabetes and cardiovascular disease at younger ages [1]. Prevention and control of these diseases and obesity were core priorities in the Sustainable Development Goals established by the United Nations in 2015 [2].

Following these guidelines, the WHO European Region launched the WHO European Childhood Obesity Surveillance Initiative (COSI). According to the results of the fifth round of data collection (2018–2020) in the WHO European Region during that period, 29% of schoolchildren aged 7–9 were overweight (overweight + obesity). Countries with the highest prevalence were the Mediterranean countries, including Spain (with a prevalence of overweight of 38.4% in boys and 39.3% in girls, and obesity of 14% in boys and 10% in girls) [3]. Conducted in 2020, one of the latest studies on overweight and obesity in children and adolescents aged 2–17 in Spain (the ENE-COVID study) found that 33.7% of boys and 26% of girls were overweight, 13.4% of boys and 7.9% of girls suffered from obesity, and 2.9% and 1.2% of boys and girls, respectively, had severe obesity. This same study analyzed the age range of preschoolers (2–5 years) in which the figures were already a cause for concern, with average percentages of overweight of 21.7% (23.3% for boys and 20.1% for girls) and obesity of 9.7% (11% for boys and 8.5% for girls) [4].

In this context, the Ministry of Health and Consumer Affairs addressed the obesity situation in our country by entrusting the Spanish Agency for Food Safety and Nutrition to implement the Nutrition, Physical Activity, and Obesity Prevention Strategy (NAOS) [5]. This policy measure dictated recommendations such as the Pilot School Reference Program for Health and Exercise against Obesity (PERSEO), aimed at promoting healthy eating and physical activity in Spanish schools [6], and the Consensus Document on Food in Educational Centers (DoCACE), which established nutritional criteria for school menus offered in educational centers, along with the recommended consumption frequencies of each food item in school menus [7]. Spanish Law 17/2011 regarding food safety and nutrition established specific measures for the school environment, ensuring that preschool educational centers (PECs) and schools promote nutrition and food education. Meanwhile, the competent authorities have been entrusted to ensure that the meals served in these centers are varied, balanced, and adapted to each age group’s nutritional needs [6].

In addition, lifestyle changes in recent decades have led many families to resort to preschool centers, including their canteens. In Spain, the latest statistical data published by the Spanish Ministry of Education, Professional Training, and Sports for the 2021–2022 school year indicated that 67.6% of public and 81.5% of private PECs have a school canteen [8]. The school canteen is thus a nutritional education tool that helps introduce Spanish children to new tastes, eating habits, and standards for optimal diet and nutrition that will accompany them for the rest of their lives. Understanding children’s dietary preferences is essential for implementing positive changes both at home and in preschool settings. This, in turn, can contribute to preventing obesity while improving children’s long-term diet habits [9]. Notably, Spanish children consume their main meal of the day (30–35% of total daily energy) in such facilities five days a week over approximately ten months a year; thus, the role of such canteens is immense in terms of its quantitative and qualitative impact on health and well-being [10]. Consequently, the demand for sustainable and healthy school canteens is steadily growing. School canteens may operate under a variety of management structures. The most frequent models in Spain are direct (they have their own kitchen) and indirect management (by contracting the service to a catering company). The management model can influence the nutritional quality of menus served.

In Spain, given the relevance of nutritional quality, numerous studies on the subject have been promoted [11,12,13,14,15,16,17], while others have focused on the culinary variety of menus served in primary and secondary school canteens [13,14,16,17,18,19,20,21]. Further studies or surveys at the national and regional level are likewise noteworthy: these include enKid [22], ENRICA [23], ALSALMA [24], ANIBES [25], ENALIA [26], ELOIN [27], ENPE [28], ALADINO [29], and EsNuPI [30,31].

However, despite the importance of establishing adequate eating habits from the preschool stage, the quality of menus served in the canteens of early childhood education centers has rarely been studied. Certain national [32,33] and international studies [34,35,36] show that the nutritional quality of the menus served in PECs does not conform to national and international recommendations, neither in energy intake nor in the contribution of macronutrients and certain vitamins and minerals to the daily recommended allowance.

Therefore, our study’s objective was to evaluate the dietary and nutritional quality of the menus served to 1-to-3-year-old children in preschool canteens of the Spanish Ministry of Defense, based on the recommendations of national and international reference organizations. We also aimed to evaluate the influence of the canteen management model.

## 2. Materials and Methods

### 2.1. Study Design and Sampling

We conducted a cross-sectional observational study on the 448 menus offered in all the 25 PEC canteens of the Spanish Ministry of Defense throughout the Spanish territory during an entire school year (except for August). In particular, they were geographically located in the north (Galicia), south (Andalusia, Canary Islands, Melilla), center (Madrid), east (Aragon, Murcia, Balearic Islands), and west (Extremadura) of Spain. The food service was awarded to the one sole company that designed the same menu for the 20 PECs that had their own kitchen under a direct management model (DM). For the remaining 5 PECs that did not have their own kitchen, a catering service was subcontracted (contracted management model, CM), which designed its own menus and prepared them in a central kitchen for later distribution. A total of 226 DM menus and 222 CM menus were studied.

The users of all these canteens, between 1 and 3 years old, were offered a complete menu consisting of a first course, a second course (usually with garnish), and dessert. The menu included a portion of white bread; water was the only beverage.

### 2.2. Evaluation of Dietary Quality

For the evaluation of the frequency of supply of food groups, we analyzed the information contained in the monthly menus (corresponding to approximately 20 school days) and the technical data sheets provided by the company awarded the contract and by the subcontracted catering company, which included the ingredients and quantities of each component used in the prepared dishes. The dishes’ main ingredients were counted as a portion of a meal, given that the portion size was adjusted to the children’s age. Once we had calculated the transformation of all dishes in the monthly menus into servings, we summed them up by food group and compared the sum with the number of servings recommended in the DoCACE [7]. We then extrapolated these weekly recommendations, corresponding to 5 school days, to monthly recommendations (20 school days) in order to compare them to other studies [33,37,38].

We also assessed the menus’ level of adherence to the following criteria in terms of the variety of school canteen menus as recommended by the PERSEO Program [6]: monthly rotation of foods for the same food group, variety of recipes (to avoid repeating the same recipe in the same week or two consecutive weeks), and variety of culinary processes (boiling, stewing, baking, grilling, and frying).

### 2.3. Analysis of Nutritional Composition and Adherence to Recommendations

We used spreadsheets and technical data sheets to determine the menus’ quantitative composition. From these data, we calculated the average amount in each serving and estimated macronutrients, micronutrients, and energy intake using the DIAL^®^ program (version 2020) for diet evaluation and food calculations. This software was designed by a group of prestigious researchers with expertise in human nutrition from the Faculty of Pharmacy of the Complutense University of Madrid in collaboration with engineers specialized in medicine and biology (Alce Ingeniería, S.L. Madrid, Spain) [39]. To evaluate the data we had obtained for energy, protein, and micronutrient content, we compared them with the nutritional recommendations for 1-to-3-year-old children as set out in the *Food Composition Tables* by Moreiras et al. [40].

To assess the adequacy of energy intake (a school lunch should provide one-third of a child’s total daily energy) and the contribution of macronutrients to total daily energy intake, we used the guidelines set out by the PERSEO Program [6]. Other indicators we used to determine diet quality were lipid profile, expressed as the percentage of energy provided by the different types of fatty acids with respect to the total energy intake, and fat quality as evaluated by several indexes or ratios among fatty acids (FA) established in the nutritional objectives of the Spanish Society of Community Nutrition (SENC) [41].

### 2.4. Statistical Analysis

We performed a descriptive analysis of the quantitative variables using central tendency, (mean) dispersion (standard deviation, SD), and frequencies. The normality of the distribution of the quantitative dependent variables was determined using the Kolgomorov–Smirnov test. The homogeneity of variances was analyzed with the Levene test. Subsequently, to determine the relationship between the dichotomous independent variable (DM/CM) and the quantitative dependent variable with a parametric distribution, we used Student’s *t*-test. The effect was assessed using the mean difference, and precision with the 95% confidence interval. A value of *p* < 0.05 was established as the degree of statistical significance when comparing the different management models. The computer application we used was the IBM^®^SPSS^®^ Statistics version 24 package.

## 3. Results and Discussion

### 3.1. Evaluation of Dietary Quality

#### 3.1.1. Frequency of Food Groups

Table 1 shows the mean supply we assessed of the different food groups after evaluating the menus and the percentage of monthly menus that met the weekly recommendations of the DoCACE [7], once extrapolated to monthly menus (20 school days). Comparing results according to the type of management, we found statistically significant differences in all food groups except precooked foods.

In the menus of educational centers, cereals and their derivatives (rice, pasta, and bread) should form the basis of children’s diets. A daily ration of bread was included in both service modalities (DM and CM). In DM canteens, over 70% of the monthly menus met the recommendations of 4 monthly pasta servings, averaging 4.2 servings/month. In addition, over 60% of menus reached the recommended frequency of 4 rice servings with a total mean of 3.6 servings/month. However, in CM canteens, rice quantity fell far short of the recommended 4 servings (1.5 servings/month) and did not meet the recommendations in any monthly menu. This deficit could be resolved by decreasing the high amount of pasta dishes (7.4 servings/month), which exceeded the recommendations in all monthly menus. The healthy eating pyramid emphasizes the importance of providing children with foods based on whole grains (bread, pasta, and rice) because they are richer in fiber, vitamins, and minerals than refined ones [42].

The other block of first courses was made up of vegetables (including potatoes) and legumes. It is recommended that they be consumed between four and eight times a month. In this case, 100% of the CM monthly menus met these requirements, averaging 5.7 monthly vegetable servings and 6.4 monthly legume servings. Meanwhile, in DM canteens, 72% of the menus exceeded the recommendations for vegetables, with an average offering of 8.8 times per month. In contrast, legumes presented a slight deficit (3.6 servings/month) in 55% of the monthly menus with regard to the recommendations.

It is recommended that children consume vegetables and tubers on a daily basis, either cooked in first courses or raw as in salads, which are the best way to digest their minerals. Therefore, it is advisable to serve between 12 and 16 varied salads per month as a garnish accompanying second courses. Salad side dishes in DM canteens almost reached this target (11.3 servings/month), given that they were present in more than half of monthly menus, whereas in CM canteens, they did not come close to the desired value, with only 6.2 servings on average; only one monthly menu fulfilled the recommended frequency.

Conversely, CM canteens presented other types of side dishes (potatoes, vegetables, legumes, etc.) more frequently (9.2 servings/month) than the recommended frequency (4–8 servings/month); the recommended frequency was thus exceeded in 64% menus per month. Seventy-three percent of the DM monthly menus complied with the reference values, with a monthly mean of 4.1 servings. The quantity of potatoes was low, and frying tended to be replaced by other forms of cooking (boiling, steaming), thus avoiding the excess of fried potatoes and the consequent high salt intake reported in the ENALIA study [26].

For the second course, meat and fish, the primary sources of protein, are recommended to be consumed 4–12 times, while eggs are used in 4–8 servings per month. In DM canteens, these requirements were met for meat and fish in all 11 monthly menus, and for eggs in all months except February, with an average of 7.2, 6.5, and 4.1 servings, respectively. However, in CM canteens, only 18% of the cycles met the established frequencies for eggs with an average of 3 servings, and only 70% for fish with an offer of 3.8 servings per month. These deficiencies could be addressed by increasing the offer of those two second courses to the detriment of meat consumption, which presented an average of 10 times per month, close to the recommended upper limit. In addition, such a reduction in meat consumption would help to adjust other macronutrient and micronutrient imbalances in the CM menus, such as excessive protein, lipid, cholesterol, and sodium intake.

It is important to remember that the DoCACE [7] recommends avoiding serving meat preparations with a higher fat content (sausages, hamburgers, meatballs, etc.) more than once a week; it also recommends avoiding fried side dishes. This aspect was usually complied with in DM and CM canteens.

On the other hand, the DoCACE [7] advises limiting the use of precooked products (cannelloni, pizzas, croquettes, dumplings, battered and breaded products such as empanadas and empanadillas, etc.) to a maximum frequency of three times per month, again avoiding fried side dishes. DM as well as CM canteens complied with this premise (0.9 and 1.2 servings/month, respectively).

Finally, in terms of desserts, it is recommended that seasonal and fresh fruit be consumed at least four times a week. All DM cycles complied satisfactorily by offering an average of 18.7 pieces of fruit per month; however, in the CM menus, the monthly recommendation of 16–20 pieces was not met in any monthly menu. Fruit was served only 11.9 times a month due to the fact that providers tended to present dairy dessert (yogurt) twice a week, whereas the recommendation is at most once a week. At least, yogurt was prioritized over other more sugary dairy products (custard, flan, etc.). In both service modalities, fruit was rotated (mainly among pear, apple, orange, and banana servings); nevertheless, other seasonal fruits would be desirable.

Other considerations, such as water being the only beverage and the use of olive oil for dressing or raw use, were observed in both food service management modalities (DM and CM).

To compare our results with those of the two studies previously conducted in Spanish PECs, we need to point out that those two studies based their calculations on the recommendations set out by the PERSEO Program [6], although Vergara et al. [33] also extrapolated the number of servings of all food groups to monthly frequencies. The amount of pasta and rice in the PEC menus evaluated by Vergara et al. [33] was slightly higher than recommended, while legumes were in line with recommendations. For those same dishes, Seiquer et al. [32] considered the distribution to be quite adequate. The latter authors also noted a daily varied supply of greens and other vegetables, mainly in raw form as salads. In contrast, in the PEC in Seville, defaults were noted in vegetables and other greens [33]. Both studies also observed lower frequencies than expected in the fruit group.

This diet deficit in fruits and vegetables was also observed in Spanish schools [14,16,17,19]. In a study conducted in schools of the Basque Country, authors observed that the vegetable side dish to be included in the menu was not served 40% of the time and that 70% of the children who were served vegetable side dishes did not eat them [14]. Limited research has examined the link between the food served and consumed in childcare centers. A cross-sectional study conducted in central North Carolina (USA) assessed the nutritional quality of meals provided to children and evaluated how much of the food served was actually eaten. The study found that children consumed between 61% and 80% of their meals, with vegetables being the least consumed (61.0%) [43]. All these studies concluded that the offer of vegetables should be increased (preferably in the first course, as it is ineffective in the side dish); they also recommend a greater quantity of fruit instead of dairy desserts.

Despite campaigns to promote the consumption of fruits and vegetables, such as a Spanish consensus consumption plan for such foods in schools [44], Spanish children still tend to reject vegetables and fruits due to factors such as taste, color, and a lack of familiarity. Our results show that a notable effort to introduce fruits and vegetables following recommendations is being made by the Spanish Ministry of Defense PEC canteens; this, in turn, helps to explain the encouraging results we observed in the areas of fiber, vitamins, and minerals. However, the frequency of fruits and salads in the CM menus could be improved, which, together with the indicated readjustment between meat and fish and eggs, could balance those menus’ caloric profiles.

On the other hand, in the PEC of Granada [32], protein foods of animal origin (meat, fish, and eggs) were served in accordance with recommendations, similar to the DM menus of our study. However, in the Seville PEC, the fish quantity was slightly below the recommended value, and meats were presented in excess [33]. An excess of meat foods and a deficit in fish and eggs was likewise observed in schools in Granada, the Basque Country, Madrid, Castilla y León, and Andalusia [13,14,16,17,19]. The authors of these studies recommended a decrease in meat and an increase in fish and eggs, the same measure we proposed for the CM menus in the current study.

We found statistically significant differences between DM and CM in the frequency of offerings for all food groups except precooked foods. In line with our results, Díez et al. [21] also found significant differences in the frequency of food groups depending on canteen management modality. However, in studies conducted in schools in Granada and Madrid [13,16], the authors found no significant differences in terms of menu quality prepared in the school’s own kitchen or by a catering service, although the latter modality offered more meat and less fish; meanwhile, eggs and fruit were in agreement with our results [16]. De Mateo et al. [19] also found no statistically significant differences according to the type of management, although the catering system offered higher dietary quality.

In our study, we took the DoCACE guidelines [7] as a reference, whereas the Andalusian PEC studies [32,33] were based on the PERSEO Program [6], as mentioned above. The most recent studies of Spanish school canteens also used the DoCACE recommendations, and several of them even developed questionnaires or proposed further recommendations designed to improve the DoCACE guidelines [18,19,21,37,44].

These considerations underscore the need to revise the DoCACE guidelines according to current scientific knowledge and to convert them into a standard combined with a monitoring and evaluation system. On the other hand, the regional heterogeneity of healthy food policies in schools needs to be regulated by applying consensus guidelines, as other authors have indicated [45,46].

#### 3.1.2. Monthly Rotation of Food; Recipes and Cooking Techniques

School canteens are an excellent venue for presenting new dishes to children; the opportunity should be used to introduce different types of foods within the same food group.

In the canteens we studied, we found that the recommendations for the monthly rotation of foods within the same group were met for meats (with a predominance of white poultry), fruit (although, as indicated above, it would be convenient to introduce more seasonal fruit), vegetables, and legumes. In this last group, lentils, chickpeas, and white beans were proposed in proportions similar to those observed in the study by Micó et al. [15].

However, a general lack of variety in fish stands out. In DM canteens, hake predominated; it was the only fish species offered in various presentations and culinary techniques in 10 of the 11 monthly menus we evaluated. January was the only month featuring the minimum recommended rotation of three different fish species (hake, halibut, and tilapia). The CM menus obtained minimum rotation by alternating hake with cod fillets, dab, and pink fish in six monthly menus. A further two months met the recommended three-fish alternation, as hake was accompanied by tuna patties and cod croquettes. However, the latter type of product may contain less than the desired amount of fish and may be considered a precooked product rather than a fish ration.

In addition, in the CM menus, tuna empanadas were one of the three dishes containing oily fish, together with pasta with tomato and tuna, and tuna omelet. Oily fish only appeared on DM menus in two months as part of a tuna omelet. This highlights the scarcity of oily fish on the menus, although the DoCACE encourages variety and alternation between oily and white fish, suggesting that they should be available in equal amounts [7]. Oily fish contain higher amounts of fat, and their more frequent inclusion in menus would provide higher amounts of ω-3 fatty acids, vitamin D, and calcium. In our sample, these valuable nutrients were present at low levels, although they are fundamental for the development of the nervous system and growth in general.

The companies designing the menus explained this observation by affirming that hake was the fish most accepted by children. Other fish cause rejection, including blue fish, such as tuna, which is dry, and sardines, which have too many bones. This aversion can be addressed by presenting boneless fish servings (loins, medallions, meatballs, or fish burgers), as long as these are not precooked dishes; moreover, vegetable-based sauces should be given priority.

All PECs in the study by Vergara et al. [33] complied with food rotation guidelines except one canteen, which offered only hake in the fish group. In studies carried out in schools, the fish rotation variety was generally considered adequate [18,21]. In particular, the latter study evaluated the culinary quality of school menus in Asturias; these authors observed that the variety of foods within food groups was well met in more than 80% of the PEC. The groups that did not meet the established variety criteria were fruits (four varieties/month) and carbohydrates (≥ six varieties/month). In the case of fruits, the variety could not really be assessed since it appeared as “seasonal fruit” subject to market availability and price. Regarding carbohydrates (pasta, rice, and potatoes), schools with DM canteens featured more variety, possibly due to the greater flexibility provided by self-management [21]. The study by De Mateo et al. [19] also observed adequate food variety.

Regarding the rotation of recipes over two consecutive weeks, we found that this was a point that required improvement in DM menus, as shown in Table 2.

In first courses, stewed potatoes were the most frequently repeated recipe, followed by pisto (sautéed vegetables), rice-based dishes (rice with tomato, three-delicious rice, rice salad), and pasta with tomato. The repetition of recipes in the second-course fish dishes was more frequent than in the meat dishes. The most frequently repeated recipes were hake in green sauce and roasted chicken hams.

Vergara et al. [33] found that five of the six PECs they studied had excessive repetition of recipes for the same group of foods, especially in terms of garnishes. Schools in Asturias complied acceptably with the recommendation to avoid repeating recipes in the same fortnight. Researchers found that compliance was better in schools with CM [21], as in our study. However, a high percentage of schools did not specify which recipe they used (indicating only general terms such as “vegetables”, “meat”, “fish”, “oily fish”, and “white fish”), which implies a limitation in the assessment of dietary quality.

In terms of culinary techniques, both management modalities featured the correct variety. The main processes they used, both in the first and second courses, were boiling, stewing, baking, grilling, and frying. In the food group of vegetables, certain months featured an excessive presence of purees, which could have been alternated with another technique, such as sautéing. In DM canteens, eggs were invariably presented as omelets with different ingredients (cooked ham, cheese, zucchini, or tuna). It would be advisable to introduce the children to other egg preparation processes, such as boiling. In the catering modality (CM), Díez et al. [21] found limitations in the variety of egg recipes and cooking techniques (potato omelet in sauce or boiled egg) due to cold transport and subsequent heating.

Vergara et al. [33] also found the correct variety of culinary techniques in the PEC menus they studied, with a greater use of boiled and baked methods to the detriment of frying.

In a study on the effectiveness of a dietary counseling system, Morán et al. [18] observed that counselors did not need to recommend menu modifications due to an insufficient rotation of culinary techniques. In schools in Castilla y León, the recommended rotation of culinary techniques was also satisfactorily fulfilled, although the proportion of fried, battered, and breaded foods was high [19]. Finally, in an evaluation of the menus served in public, private, and charter schools, Castro et al. [17] concluded that the variety of techniques used in the preparation of recipes should be expanded.

### 3.2. Analysis of Nutritional Composition and Fulfillment of Recommendations

#### 3.2.1. Energy, Macro- and Micronutrient Intake

Table 3 shows the mean (±SD) energy, macronutrient, cholesterol, and fiber content of the menus we evaluated throughout the school year in the Spanish Ministry of Defense PEC canteens, according to management model.

The recommended energy intake for children aged 1 to 3 years is 1250 kcal/day for moderate activity, increasing by 20% for high activity and decreasing by 10% for light activity [40]. The midday meal offered in school canteens should provide about 35% of the total daily energy requirement [6]. Considering this proportion and the recommendations for moderate physical activity, the menus prepared in the DM kitchens, with an average contribution of 476.7 kcal, and those served in the CM canteens, with a contribution of 449.0 kcal, met the recommended energy requirements with a contribution of 38.2% and 35.9% of total daily energy, respectively.

In a study carried out in preschools in Granada, Seiquer et al. [32] found an estimated average intake of 512.5 kcal/day, which was 30.1% of the recommendations on which they were based (1700 kcal/day for 4-to-6-year-old children). In another study in Seville preschools, the menus provided 30.6% of energy with respect to the reference recommendations (1300–2000 kcal/day for 1-to-3-year-old children) [33]. In schools for older children, energy values similar to our study have been observed, lying between 35.0 and 38.0% [11,12,13]. The latter authors justified the slightly elevated energy content by the high contribution of proteins and lipids to the detriment of carbohydrates.

Both management models in the canteens in our study served a high amount of protein (DM, 18.3 g and CM, 19.6 g) when compared with the daily intakes recommended by Moreiras et al. [6] (23 g for 1-to-3-year-old children), especially considering that children will tend to consume three or four more meal rations throughout the day. Regarding the energy distribution of macronutrients in the main meal of the day, it is generally recommended that protein content should contribute 12–15% to total caloric intake [6]. This was followed in DM menus (14.5%), and we only observed a slight excess in CM menus (17.6%). The protein excess in our study is in line with that observed in the two previous studies carried out in Spanish PECs. Both Seiquer et al. [32] and Vergara et al. [33] observed a high protein contribution to diet (more than 17%). Similar values, even higher ones, were found in evaluations carried out in schools in Madrid [11,16], Tenerife [12], Granada [13], Vizcaya [14], Valencia [15], and Seville [32]. The high protein content was due to the excessive consumption of foods of animal origin (mainly lean meat, white fish, and eggs); this has been the pattern generally observed in industrialized countries in recent years. It is important to adjust the amounts to children’s nutritional needs at this stage. One possibility would be to reduce the portion sizes of protein foods, especially meat. In the case of DM menus, the frequency of consumption of legumes should be increased, as they do not reach the recommended minimum, and meat consumption should be reduced. If proteins are in excess, there is a greater risk of childhood obesity and a greater environmental impact; moreover, proteins can displace the consumption of other healthy foods [47].

Regarding carbohydrates, there are no recommended intakes (RI), but nutritional targets advise that 50–60% of total energy should come from carbohydrates [6]. This recommendation was met by the DM menus (53.6%) but was lower than recommended in the CM menus (44.7%). The amount of carbohydrates could be increased by eating more vegetables, salads, and fruit, as these are foods that do not reach the monthly recommendations in the case of CM menus. If we compare our results in DM canteens with those obtained in other PECs, we see that they are similar to those reported by Vergara et al. [33], who found contributions of 50–60% of the recommended amount, and by Seiquer et al. [32] with 52%. In schools, Campos et al. [12] and Micó et al. [15] also reported an adequate carbohydrate intake (54%). However, other studies in schools found a deficient carbohydrate intake similar to our results in CM menus [11,13,14,17,35]. A nutritional study carried out in Spain on 1-to-10-year-old children, the Estudio Nutricional en Población Infantil Española (EsNuPI), showed that about half of respondents did not reach the recommendations [48]. In both modalities, the nutritional targets for simple sugars (<10% total daily energy) were slightly exceeded [41], with values of 13.9% and 10.6% in DM and CM, respectively. It must also be considered that taste preference, especially for sweet and fatty tastes, is one of the factors that affect children’s food intake and eating habits, and consequently it must be taken into account that the consumption of a great percentage of calories from added sugars could be a strong risk factor for increased weight and/or obesity in children [49]. Ever since the early 2000s, the WHO has highlighted the importance of a high intake of complex carbohydrates, as they are the primary energy source in the human diet. Foods such as cereals (bread, pasta, and rice), potatoes, fruit, and vegetables provide these macronutrients, in addition to micronutrients and fiber, and constitute an advantage over simple sugars by preventing childhood cariogenesis and obesity while also preventing chronic disease in adults [50,51].

Regarding fiber, the mean intake in our study was 7.83 g in DM and 7.04 g in CM. In two previous studies on PECs in Andalusia, authors observed a fiber intake close to 8 g/day [32,33], similar to the fiber intake found in school menus [12,13]. Following AHF (American Health Foundation) recommendations, the American Academy of Pediatrics (AAP) proposes, for children and adolescents aged 2 to 20, a minimum intake of dietary fiber equivalent to the child’s age plus 5 g of dietary fiber per day, suggesting a safe range of dietary fiber intake between age plus 5 and age plus 10 g/day [52]. In its Dietary Reference Intakes (DRI), the US National Academy of Sciences (NAS) recommends an adequate intake (AI) of dietary fiber for children aged from 12 months to 18 years of 14 g of total fiber per 1000 kcal per day (14 g/1000 kcal × average energy intake/d) [53]. For children under 3 years of age, the European Food Safety Authority (EFSA) established an adequate intake of 10 g of fiber per day, while the WHO suggests an intake of naturally occurring dietary fiber consumed in foods of at least 15 g of fiber per day for 2-to-5-year-old children [51]. Considering that our results refer to only one of the day’s meals, they can be regarded as high. Excess fiber intake can harm the absorption of certain vitamins and minerals, although this can be compensated by the benefits fibers provide in terms of weight control, cholesterol, glycemia, etc.

The recommended contribution of lipids to total energy should not exceed 30–35% [6]. In the PEC canteens we studied, the energetic contribution of fat to the diet was in accordance with the recommendations in the DM menus at 31.9%, whereas those of the CM menus exceeded recommendations by contributing 37.7%. In the menus of the PEC in Seville, the total fat was below the recommendations [33]. In contrast, the menus of the PEC in Granada almost reached 34% fat [32]. In studies carried out in schools, certain results fall below the recommendations [12], others comply with recommendations [14], and others exceed them [11,13], even reaching values, in one case, of around 50% in half of the menus studied [17].

In our study, the mean cholesterol intake values were 90.3 mg in the DM menus and 59.3 mg in the CM menus. As we are dealing here with one of several daily meals, these values did not obey the nutritional objective of not exceeding a cholesterol intake of 100 mg/1000 kcal [41]. Our results agree with the mean content found in the menus of the PEC in Granada, which exceeded the recommended values by slightly over 20% [32], and with those found by Martínez et al. [13] in schools in Granada (179 mg/1000 kcal). However, Vergara et al. [33] observed an adequate cholesterol intake in all the menus they evaluated in the PEC of Seville, as did Campos et al. [12] in schools in Tenerife. The elevated cholesterol levels in our study are associated with high fat levels in the CM menus. Therefore, in addition to the priority measure of reducing fat intake, the dietary restriction of cholesterol should also be considered. Despite the important biological functions of cholesterol for the body, a high cholesterol intake can negatively affect a child’s cardiovascular health. For this reason, the frequency of meat consumption could be reduced, and the frequency of fish consumption could be increased, as they are generally lower in fat and cholesterol. Another possibility would be to offer more fruit for dessert in the case of CM menus, instead of other alternatives such as dairy desserts.

As can be observed in Table 4, most mean vitamin intake rates were adequate, and some even exceeded the daily RI proposed by Moreiras et al. [40]. This was the case for niacin, vitamin A, and vitamin K in both management modalities. Vitamin D contents were particularly low in the menus of both management types (DM: 2.1%; CM: 1.4% of the daily RI). Another vitamin that presented low values in our study was vitamin E in the CM menus, with 26.5% of the daily RI. However, the intake of this vitamin could be increased by including more green leafy vegetables in the first courses, and nuts as an alternative to dessert or in the preparation of certain dishes. The EsNuPI study also observed a vitamin D deficiency that could be considered a cause for concern [30]. Vitamin D is important for preventing osteoporosis, cardiovascular disease, type 2 diabetes, and cancer. This deficiency can be compensated with the other daily meals and other vitamin D sources such as dairy products (two servings/day), egg yolk and oily fish, or even fortified formulas. In addition, there is the possibility of endogenous synthesis of this vitamin by exposure to sunlight.

Our results are consistent with those of other Spanish PECs, where an adequate vitamin content was observed in canteen menus [32,33]. Moreover, in the case of the Grenadian PEC [32], certain vitamins (folic acid, vitamins A and C) exceeded the DRI, which in turn agrees with the results of the meals assessed in other schools [11,12,13,14]. Contrary to our study and those cited above, vitamin D exceeded the DRI in the PEC in Seville. In contrast, the latter authors found amounts of vitamin C [35] and E much lower than recommended [33].

As for mean intakes of minerals (Table 5), they were generally in line with the DRI for preschool children proposed by Moreiras et al. [40], although certain micronutrients exceeded recommendations. That excess was observable in potassium, sodium, and selenium in the menus of both modalities (DM and CM). On the other hand, the minerals that presented the lowest levels of DRI were calcium (DM: 17.8%; CM: 21.2%) and zinc (DM: 20.8%; CM: 20.7%). Our results are in accordance with those assessed in other PECs in Spain. Vergara et al. [33] found high potassium and selenium intakes, as well as a low calcium intake. Seiquer et al. [32] reported calcium levels of 16% of the DRI and 21% in zinc. In schools, Campos et al. [12] found deficient levels of iodine, zinc, and iron, whereas Del Pozo et al. [11] observed that all the minerals they evaluated (calcium, iron, magnesium, and zinc) contributed more than 35% to DRI.

Zinc deficiency has been related to growth deficits and alterations in taste and smell; thus, it is necessary to promote the consumption of the primary sources of this mineral (meats, fish, legumes, dairy products, and cereals) and to complement their consumption in other meals of the day. This would help to bring the intake of this micronutrient, essential in the synthesis of thyroid hormones and in the child’s cognitive capacity, closer to the recommended intake. Low calcium levels can be explained by the fact that the highest intake is provided by dairy products consumed during other meals of the day. Thus, the DRI for calcium, equivalent to two servings of dairy products/day, can be compensated with breakfast or a mid-afternoon snack. In the menus served in canteens of both management models, the consumption of legumes, nuts and green leafy vegetables can be promoted, as they are also a source of calcium. This would ensure an adequate intake of this mineral, essential for the optimal formation of bone mass and skeleton growth in children. In relation to ossification, phosphorus intake must also be considered; a Ca/P ratio between 1:1 and 2:1 is recommended in diet, as such a ratio ensures the maximum utilization of calcium [48].

This ratio was not reached in any of the menus we studied (DM: 0.40; CM: 0.41). As most menus tended to have a high protein content, the calcium content was low and phosphorus content was high, similar to two other studies in PECs [32,33] and in the Spanish child population aged 1 to 10 [30]. This imbalance may adversely affect bone formation and development at preschool age and may lead to a risk of osteoporosis in the future. Adequate iron values were obtained in the Andalusian PEC and in our study; iron requirements are elevated in high growth phases, and iron intake is usually ensured with meat servings.

Regarding salt intake, the Spanish Association of Pediatrics recommends 0.8 g/day of sodium for children aged 1 to 3 years [54]. The mean sodium intake in the DM menus in our study exceeded the daily recommendation with a mean amount of 805.8 mg; in CM menus, it practically reached the DRI, with an intake of 767.9 mg. This excessive amount of sodium could be due to the high consumption of precooked foods, chips, meat, meat products, and fish and fish products. Still, in our study, a discretionary use of salt (during cooking and, to a lesser extent, at the table) should be considered. In this context, another nutritional index for the determination of diet quality is the sodium/potassium ratio, which should be close to 1 [55]. We found high sodium values, but potassium intake was also high; therefore, this index was in accordance with the recommendations due to an excess intake of both minerals (DM 0.99 and CM 0.95).

#### 3.2.2. Quality Indicators

The results of our evaluation of menu quality per various indicators, such as calorie profile, lipid profile, and fat quality, are shown in Table 6 according to management model.

Regarding quality indicators, the caloric profile of the DM menus was in line with the established nutritional objectives. However, the CM menus were imbalanced, with an excessive consumption of fats and proteins coupled with an insufficient intake of carbohydrates, which is in line with what has been found in previous studies of PEC canteens [11,12,13]. The EsNuPI study confirms this dietary pattern (protein 16.5%, carbohydrates 45.4%, and fat 36.5%) [28], similar to other studies on Spanish children and adolescents: ALSALMA [24], ANIBES [25], and ENALIA [26]. Readjustment in the CM menus assessed in our study could be achieved by decreasing meat servings and increasing the serving quantities of fish, eggs, and plant-based foods (salads and fruits).

A balanced lipid profile is known to be beneficial in preventing cardiovascular disease because of the protective role played by unsaturated fats and the association between an excessive intake of saturated fatty acids (SFA) and high plasma low-density lipoproteins cholesterol (LDL-C) levels.

In a systematic review of the health effects of saturated and trans-fatty acid intake on children and adolescents [56], a reduced intake of saturated fatty acids (SFA) resulted in a decrease in total cholesterol, LDL-C, and diastolic blood pressure. A small number of trials indicated that the most pronounced effect was obtained when SFA were replaced mainly by polyunsaturated fatty acids (PUFA) or by a mixture of PUFA and monounsaturated fatty acids (MUFA) and when SFA intake was reduced to less than 10% of the total fat intake. There were no significant effects on other outcomes and no indications that reducing SFA intake had adverse effects [57].

Therefore, in addition to the amount of total fat, it is important to ascertain which type of SFA was ingested. In the menus we assessed, the percentage of SFA approached the nutritional targets of 7–8% (DM: 6.8% and CM: 8.7%). A study conducted in Georgia (USA) also found high levels of SFA [35]. The percentage of total energy contributed by PUFA also generally approached the nutritional targets of 5%, with 4.7% and 4.9% in the DM and CM menus, respectively. The nutritional target of 20% MUFA in the DM menus was unmet (17.3%). This target was slightly exceeded in the CM menus (20.7%), possibly due to a greater use of olive oil in recipes common in the Mediterranean diet. In the Seville PEC study, there were deficient values for unsaturated fats (MUFA and PUFA) and adequate values for saturated fats [33], which agree with the results in school menus [9,10]. Conversely, the menus of the PEC in Granada presented a correct proportion of MUFA with respect to total energy [32].

Knowing the average fat intake and the lipid profile is relevant, but it is also important to consider fat quality. Thus, the nutritional objectives for energy intake from trans-fatty acids indicate that they should be less than 1%. In the menus we assessed, this was the case in both modalities (DM: 0.40%; CM: 0.50%). The mean intake of PUFA ω-3 was lower than the recommendation of 1–2% of total energy in the menus of both managements: DM 0.7% and CM 0.7%. The values of PUFA ω-6 were 3.9% in the DM menus and 4.1% in those of CM, which lay around the 3% recommended by Moreiras et al. [40] and Serra and Aranceta [41]. High-income countries usually have a diet with a high content of ω-6 PUFA to the detriment of ω-3 intake; thus, the ratio between ω-6/ω-3 usually exceeds the advised ratio (<4:1), as occurred in our study (DM 6.31 and CM 6.93). Two other ratios habitually used to assess fat quality are PUFA/SFA and (PUFA + MUFA)/SFA. For the first ratio, the nutritional objective establishes that the index should be ≥0.5; in our study, it was fulfilled in the menus of both modalities (DM 0.74 and CM 0.60). The recommendation for the (PUFA + MUFA)/SFA index establishes that it should be ≥2. This was followed in both the DM and CM menus. In the study of the menus served in the PEC in Granada, similar results were obtained for both indexes: PUFA/SFA 0.8 and (PUFA + MUFA)/SFA ≥ 2 [32]. The EsNuPI study shows a high intake of lipids and SFA, coupled with a low intake of essential amino acids and PUFA ω-3, resulting in a high ω-6/ω-3 ratio [31]. A good intake of essential amino acids as well as ω-3 and ω-6 PUFA is important for child development and future health as an adult.

Finally, for our quantitative analysis of energy, nutrients, and other menu quality indicators, we used a nutritional computer program. Food composition tables used in research studies vary to a great extent; the same applies to dietary computer programs, as pointed out by Lupiañez-Barbero et al. [58]. This leads to biased and non-comparable results. Another controversial issue is the reference recommendations for measuring energy intake in school menus. In our study, we opted for the nutritional recommendations published in the *Food Composition Tables* book [40] due to its broad coverage and its overview of the needs of the Spanish population; this successful manual has gone through 20 editions.

Since school menus play an essential role in covering part of children’s daily energy and nutrient requirements, and bearing in mind that they should be complemented with the remainder of the meals consumed in the family home, an evaluation of nutritional risks should consider the adequacy of diet for the correct growth and development of a country’s child population. Our results reveal a margin for improvement in managing these risks in certain menus, allowing for an adjustment of caloric and lipid profiles and micronutrient intake. Additionally, since daycare centers support children during their early years, they have the opportunity to influence lifelong eating habits and encourage a responsible attitude toward health.

## 4. Conclusions

In terms of dietary quality, the CM menus showed a relatively low supply of foods such as rice, eggs, fish, vegetables, salads, and fruits. In both modalities, the fish group was the one that rotated the least, with a predominance of hake and a lack of oily fish. In terms of recipe variety and culinary techniques, the menus we studied were generally in line with recommendations for school canteens.

Regarding nutritional quality, the menus’ energy values were correct; however, the energy profile in the contracted management menus was higher than recommended in protein and fat, and lower than recommended in carbohydrates, coinciding with other studies conducted in Spain. The lipid profile was adequate, with high values of cholesterol, simple sugars, and fiber, whereas it was deficient in ω-3 fatty acids. Average daily vitamin and mineral intakes, on the whole, were adequate. However, certain imbalances stood out: an inadequate calcium/phosphorus ratio, a low intake of vitamin D, iodine, and zinc, and, finally, an excessive intake of vitamin K, selenium, potassium, and sodium. With respect to the influence of the canteen management model, in general terms, the nutritional qualities of DM menus were better than those of the CM model, particularly regarding the caloric (protein, carbohydrate, and lipid) and SFA profiles, as well as PUFA/SFA, PUFA + MUFA/SFA, and ω-6/ω-3.

The findings of this study could be applied to other Spanish canteens of preschool centers, since their diet pattern is mostly similar. Nevertheless, more studies are required and the standards in Spain need to be unified in order to allow for the comparison of results among studies; this applies more specifically to studies of preschool canteens. The results we obtained could thus help to standardize criteria in order to establish or legislate nutritional reference values and single limits, thus allowing researchers to obtain comparable data in the preschool population and to implement nutritional intervention programs in PEC canteens, thus working toward the adoption of a more balanced dietary pattern.

## Figures and Tables

**Table 1:** Food group supply (mean of servings/month) and percentage of monthly menus meeting DoCACE recommendations [7] according to the management model.

		DM Canteens		CM Canteens		
Food Group	DoCACE Recommendations (Monthly Servings)	Mean (SD) Monthly Servings	Percentage (%)	Mean (SD) Monthly Servings	Percentage (%)	*p* Values
**First courses**						
Rice *	4	3.64 (0.50)	63.60	1.45 (0.52)	0.00	<0.001
Pasta *	4	4.18 (0.75)	72.80	7.36 (0.67)	0.00	<0.001
Legumes *	4–8	3.55 (0.82)	54.50	5.73 (1.10)	100.00	<0.001
Vegetables * (including potatoes)	4–8	8.82 (1.08)	18.20	6.36 (0.50)	100.00	<0.001
**Second courses**						
Meat *	4–12	7.18 (1.40)	100.00	10.0 (0.89)	100.00	<0.001
Fish *	4–12	6.45 (1.04)	1000.00	3.82 (0.60)	72.70	<0.001
Eggs *	4–8	4.09 (0.54)	909.00	3.00 (0.63)	18.20	<0.001
**Precooked foods**	0–3	0.90 (0.57)	100.00	1.18 (0.75)	100.00	0.226
Garnish *						
Salads *	12–16	11.30 (3.17)	54.50	6.18 (0.87)	9.09	<0.001
Other ^a^*	4–8	4.09 (1.14)	72.70	9.18 (1.33)	63.60	<0.001
**Desserts**						
Fresh fruit *	16–20	18.70 (1.56)	100.00	11.90 (0.54)	0.00	<0.001
Other ^b^*	0–4	0.73 (0.79)	100.00	7.64 (0.67)	0.00	<0.001

^a^ Potatoes, vegetables, legumes, etc. ^b^ Milk, yogurt, fresh cheese, curds, nuts, natural juices, etc. * Statistically significant differences (*p*-value < 0.05). Student’s *t*-test was used to calculate differences between management models. DM: direct management model; CM: contracted management model.

**Table 2:** Number of non-compliances due to recipe repetitions between two consecutive weeks in DM canteens.

		DM Canteens	
Food Group	1st–2nd Week	2nd–3rd Week	3rd–4th Week
Legumes, pasta, and rice	7	6	5
Meat and meat products	2	2	1
Fish	3	2	4

DM: direct management model; CM: contracted management model.

**Table 3:** Energy and macronutrient content of complete menus, according to management model.

Nutrient	DM (n = 226)	CM = (n = 222)	*p* Values
	Mean (SD)	Mean (SD)	
Energy (kcal) *	476.70 (52.50)	449.00 (51.40)	<0.001
Proteins (g) *	18.40 (3.78)	19.60 (3.91)	0.001
Fat (g) *	17.90 (4.64)	18.90 (4.39)	0.021
Cholesterol (mg) *	90.30 (71.60)	59.30 (34.50)	<0.001
Carbohydrates (g) *	56.10 (8.32)	46.60 (8.54)	<0.001
Fiber (g) *	7.82 (2.02)	7.04 (2.85)	<0.001

*: Statistically significant differences (*p*-value < 0.05). Student’s *t*-test was used to calculate differences between management models. DM: direct management model; CM: contracted management model.

**Table 4:** Vitamin content (mg or µg) of the complete menus, according to management model.

Vitamin	DM (n = 226)	CM (n = 222)	DRI ^1^	*p* Values
	Mean (SD)	Mean (SD)		
Thiamine (mg)	0.32 (0.13)	0.34 (0.15)	0.5	0.166
Riboflavin (mg)	0.29 (0.07)	0.30 (0.08)	0.8	0.472
Niacin (mg)	8.64 (2.06)	8.48 (2.46)	8	0.445
Pyridoxine (mg)	0.57 (0.21)	0.59 (0.21)	0.7	0.508
Cobalamin (µg)	0.77 (0.44)	0.82 (0.61)	0.9	0.272
Folates (µg) *	90.60 (33.90)	81.90 (45.80)	100	0.022
Ascorbic acid (mg) *	46.90 (20.80)	29.30 (19.00)	55	<0.001
Vitamin A (µg) *	389.50 (280.40)	335.60 (219.40)	300	0.024
Vitamin D (µg) *	0.31 (0.42)	0.21 (0.28)	15	0.001
Vitamin E (mg) *	2.21 (0.72)	1.59 (0.53)	6	<0.001
Vitamin K (µg) *	62.10 (43.60)	43.40 (28.90)	30	<0.001

^1^ DRI: Daily Recommended Intakes for children from 1 to 3 years old, by Moreiras et al. [40]. *: Statistically significant differences (*p*-value < 0.05). Student’s *t*-test was used to calculate differences between management models. DM: direct management model; CM: contracted management model.

**Table 5:** Mineral content (mg or µg) of the complete menus, according to management model.

Mineral	DM (n = 226)	CM (n = 222)	DRI	*p* Value
	Mean (SD)	Mean (SD)		
Calcium (mg) *	107.10 (32.68)	127.00 (58.40)	600 ^1^	<0.001
Iron (mg) *	3.70 (0.90)	3.52 (0.99)	7 ^1^	0.047
Iodine (µg)	21.50 (8.39)	20.00 (23.80)	55 ^1^	0.380
Magnesium (mg)	74.70 (18.10)	72.10 (14.90)	125 ^1^	0.105
Zinc (mg)	2.08 (0.69)	2.07 (0.59)	10 ^1^	0.993
Sodium (mg)	805.80 (167.40)	767.90 (307.80)	800 ^2^	0.107
Potassium (mg)	894.00 (264.70)	895.50 (286.70)	800 ^1^	0.986
Phosphorus (mg) *	263.80 (51.30)	299.40 (75.70)	400 ^1^	<0.001
Selenium (µg)	30.90 (13.40)	29.40 (14.40)	20 ^1^	0.278

^1^ DRI: Daily Recommended Intakes for children from 1 to 3 years old, by Moreiras et al. [40]. ^2^ DRI: Daily Recommended Intakes for children from 1 to 3 years old, by the Spanish Pediatric Association [54]. *: Statistically significant differences (*p*-value < 0.05). Student’s *t*-test was used to calculate differences between management models. DM: direct management model; CM: contracted management model.

**Table 6:** Indicators of the quality of complete menus (calorie profile, lipid profile, and fat quality), according to management model.

Quality Indicator	DM (n = 226)	CM (n = 222)	NO	*p* Value
	Mean (SD)	Mean (SD)		
Proteins (%) *	14.50 (2.41)	17.50 (3.26)	12–15 ^1^	<0.001
Carbohydrates (%) *	53.60 (7.14)	44.70 (6.89)	50–60 ^1^	<0.001
Sugars (%) *	13.90 (3.94)	10.60 (2.75)	<10 ^2^	0.001
Fat (%) *	31.90 (7.04)	37.70 (6.45)	30–35 ^1^	<0.001
SFA (%) *	6.82 (2.30)	8.73 (2.58)	7–8 ^2,3,4^	<0.001
MUFA (%) *	17.30 (4.31)	20.70 (3.88)	20 ^2,3,4^	<0.001
PUFA (%)	4.68 (1.14)	4.86 (1.00)	5 ^2,3,4^	0.087
PUFA ω-3 (%)	0.73 (0.29)	0.68 (0.29)	1–2 ^2^	0.066
PUFA ω-6 (%) *	3.86 (1.05)	4.09 (0.97)	3 ^2^	0.020
trans-FA (%)	0.40 (0.59)	0.50 (0.53)	<1 ^2^	0.059
ω-6/ω-3 ratio *	6.31 (3.06)	6.93 (2.78)	<4:1 ^2^	0.024
PUFA/SFA *	0.74 (0.24)	0.60 (0.19)	≥0.5 ^2^	<0.001
PUFA + MUFA/SFA *	5.32 (0.83)	4.95 (0.84)	≥2 ^2^	<0.001

SFA: saturated fatty acids. MUFA: monounsaturated fatty acids. PUFA: polyunsaturated fatty acids. ω: omega. FA: fatty acids. NO: nutritional objectives. ^1^ PERSEO Program recommendations [6]. ^2^ Nutritional objectives per the Spanish Society of Community Nutrition [41]. ^3^ Nutritional objectives by Moreiras et al. [40]: AGS < 7–8%; AGMI 20%; AGPI 5%. ^4^ Nutritional objectives by Ortega et al. [53]: AGS < 10%; AGMI 15–20%; AGPI 4–10%. *: Statistically significant differences (*p*-value < 0.05). Student’s *t*-test was used to calculate differences between management models. DM: direct management model; CM: contracted management model.

## Data Availability

Data are contained within the article.
